# Multimodality 3D Superposition and Automated Whole Brain Tractography: Comprehensive Printing of the Functional Brain

**DOI:** 10.7759/cureus.1731

**Published:** 2017-09-29

**Authors:** Sanjay Konakondla, Cameron J Brimley, Jesna Mathew Sublett, Edward Stefanowicz, Sarah Flora, Gino Mongelluzzo, Clemens M Schirmer

**Affiliations:** 1 Neurosurgery Department, Neuroscience Institute, Geisinger Health System; 2 Neurosurgery Department, Neurosciences Institute, Geisinger Health System; 3 Department of Radiology, Geisinger Health System; 4 Department of Radiology and Neuroradiology, Geisinger Health System

**Keywords:** 3d printing, technical skill rehearsal, patient engagement, valued care, informed consent, medical education

## Abstract

Whole brain tractography using diffusion tensor imaging (DTI) sequences can be used to map cerebral connectivity; however, this can be time-consuming due to the manual component of image manipulation required, calling for the need for a standardized, automated, and accurate fiber tracking protocol with automatic whole brain tractography (AWBT). Interpreting conventional two-dimensional (2D) images, such as computed tomography (CT) and magnetic resonance imaging (MRI), as an intraoperative three-dimensional (3D) environment is a difficult task with recognized inter-operator variability. Three-dimensional printing in neurosurgery has gained significant traction in the past decade, and as software, equipment, and practices become more refined, trainee education, surgical skills, research endeavors, innovation, patient education, and outcomes via valued care is projected to improve. We describe a novel multimodality 3D superposition (MMTS) technique, which fuses multiple imaging sequences alongside cerebral tractography into one patient-specific 3D printed model. Inferences on cost and improved outcomes fueled by encouraging patient engagement are explored.

## Introduction

The use of x-rays (XR), computed tomography (CT), and magnetic resonance imaging (MRI) has been the standard for neurosurgical education, planning, and treatment. Although these have contributed significantly to advancing accuracy and efficacy of neurosurgical practice, these modalities have limitations in their two-dimensional landscape. Functional regions, previously based on interpretations relative to structural zones, have been restructured and redefined with the advent of automated whole-brain tractography (AWBT), functional magnetic resonance imaging (fMRI), and invasive intraoperative cortical and subcortical stimulation [1-2]. Intraoperative neuronavigation helps to appreciate not only eloquent areas but also critical neurovascular structures within or in proximity to the surgical areas of interest. 

Collating and translating these two-dimensional (2D) images into an intraoperative three-dimensional (3D) space can be a challenging task and comes with an appreciated degree of variability between surgeons. We have developed tools to aid us in the operating room but continue to lack a simple haptic representation of the anatomy of interest that can be used before a procedure. With conventional preoperative imaging modalities attempting to display spatial 3D structures viewed on a 2D screen, conceptualizing the 3D properties of the intracranial environment remains subject to error. Intraoperative mapping techniques and neuronavigation require real-time mapping of the brain, subsequently necessitating real-time surgical decision-making, which is less than optimal.

Additive manufacturing or 3D printing initially emerged in the 1980s and has since been utilized in multiple industries, including medical, aviation, automotive, and many other fields [3]. As 3D imaging technology, computer-aided design software, and additive manufacturing were more readily available and affordable, they became commonly applied in neurosurgery. Neurosurgical targets, along with their relevant surrounding neurovascular structures, can be plotted and crafted into 3D models that can be utilized for a myriad of possibilities, including preoperative planning, resident training, and patient education. Three-dimensional models have been employed for examination of bony structures, aneurysms and arteriovenous malformations, tumors, and ventricular systems.

In this report, we describe a novel multimodality 3D superposition (MMTS) technique, which incorporates relevant imaging data layered onto neighboring white matter tracts yielded from our tractography software all onto a single 3D printed model. We demonstrate its utility and benefits for preoperative planning, training, and education. Implications for improved outcomes and targeted valued care are explored.

## Technical report

Software and protocols

As part of the institutional preoperative imaging protocol, patients are imaged on a 1.5 Tesla (T) Siemens Aera (Siemens, Plano, TX) or 3T GE Discovery mr750 (GE Healthcare, Chicago, IL) MRI unit. Imaging sequences include 3D isotropic post-contrast T1-weighted spoiled gradient echo sequences magnetization prepared rapid acquisition gradient echo (MPRAGE) for Siemens, spoiled gradient recalled (SPGR) for GE, and diffusion tensor imaging using 32 diffusion directions. For patients in whom functional magnetic resonance imaging (fMRI) is desired, task-based blood oxygen level dependent (BOLD) fMRI is performed using selected motor and language paradigms following patient training and education by the supervising neuroradiologist and by using an Invivo ESys display unit (Invivo, Gainesville, FL).

Pre- and post-processing of fMRI data is performed using an Invivo Dynasuite Neuro 3.0 workstation (Invivo, Gainesville, FL) with statistical thresholding of the fMRI probability maps performed by the neuroradiologist. Tractography is currently performed semi-automatically using Dynasuite’s commercial deterministic fiber tracking algorithm, regions of interest (ROIs), fractional anisotropy (FA), and curvature thresholding. The thresholding is performed by adjusting FA and curvature values and by visually inspecting output to maximize representation of the relevant fiber tracts and to minimize erroneous adjacent tracts. As white matter tracts are often affected by lesions and edema, FA values and curvature thresholds have proven to vary and we have not established standardized values that produce optimal results for all patients. Therefore, for fibers adjacent to surgical lesions, FA and curvature thresholds are selected to favor sensitivity in defining a tract over specificity. This process of optimal fiber tractography at our institution has not been automated. Co-registration of the anatomic T1-weighted imaging dataset with the surgically relevant fibers (a visually thresholded directionally-encoded color FA map) and fMRI-generated activation maps are subsequently performed using the Dynasuite software package.

Multimodality 3D superposition

Once the post-processing of the selected data sets is completed, the results are exported in digital imaging and communication in medicine (DICOM) format to specialized medical 3D printing software called Mimics Innovation Suite (Materialise, Leuven, Belgium). Several data sets will be fused together to allow for the various imaging studies to be included on one 3D printed model.

The brain produced from the T1 3D data is used as the anatomical base, and the other data sets are assembled on and fused with the brain. Based on the clinical application, this can include diffusion tensor imaging (DTI) fiber tracts, fMRI, computed tomographic angiography (CTA), or magnetic resonance angiography (MRA) datasets. All files produced are done in conjunction with 3D lab technologists, neuroradiologists, and neurosurgeons to ensure that the anatomy of interest is included and accurate.

**Figure 1:**
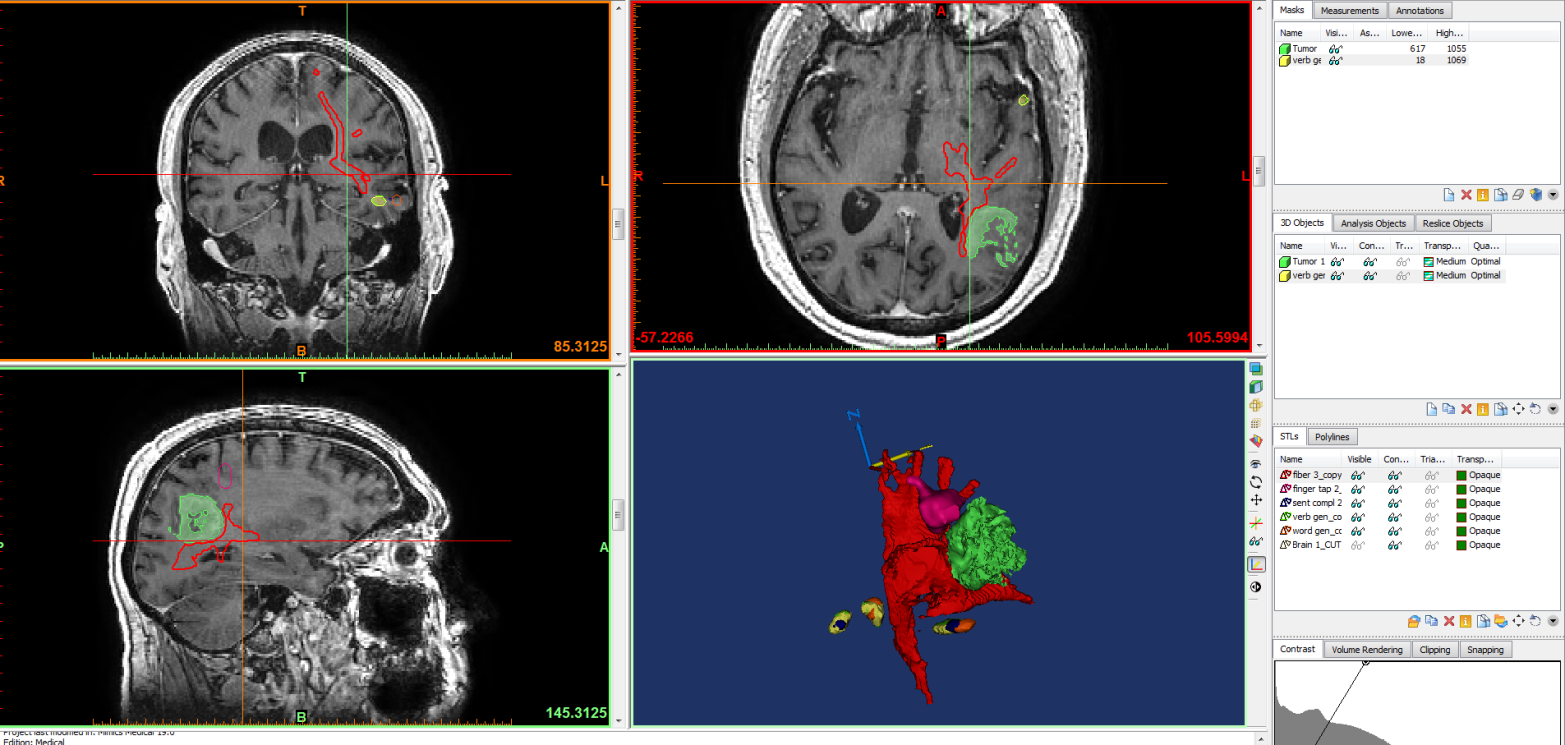
Multimodality 3D superposition T1 three-dimensional (3D) data is used as the anatomical base, and the data sets yielded from functional magnetic resonance imaging (fMRI) and tractography are assembled on and fused with the brain; tumor (green), fiber tracts (red), fMRI data (pink, yellow). Blue and yellow arrows are arrows of axes. Blue "N" represents "north direction."

**Figure 2:**
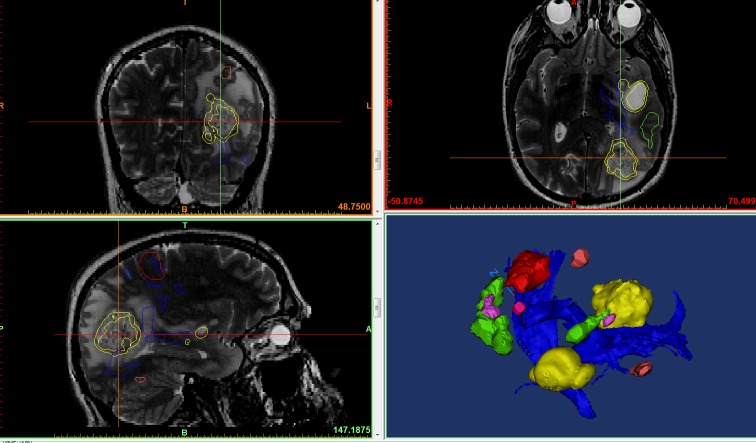
Multimodality 3D auperposition Using magnetic resonance imaging (MRI) for anatomical base, we overlay three-dimensional (3D) objects representing the tumor (yellow), fiber tracts (blue), and functional magnetic resonance imaging (fMRI) data (red, pink, green) into one model in preparation for printing.

In addition to the post-processing performed on the Dynasuite Neuro workstation, segmentation of DICOM data is performed using the Mimics Innovation Suite. For neurosurgical applications, the brain must be segmented to create a frame for adherence of the fMRI, DTI, or vessel data sets. The brain is segmented using a selected threshold value that matches white matter, gray matter, or both, and then fine-tuned with various free-hand tools (Figures 1-2). Prior to printing, the file is checked for quality using Materialise 3Matic (Materialise, Leuven, Belgium). This software can perform an advanced analysis of the model to ensure that it will successfully print. The final quality control check is done by overlaying the stereolithography (STL) file on the base T1 3D MRI scan to verify the accuracy of segmentation and fusion. 

Printing selection

**Figure 3:**
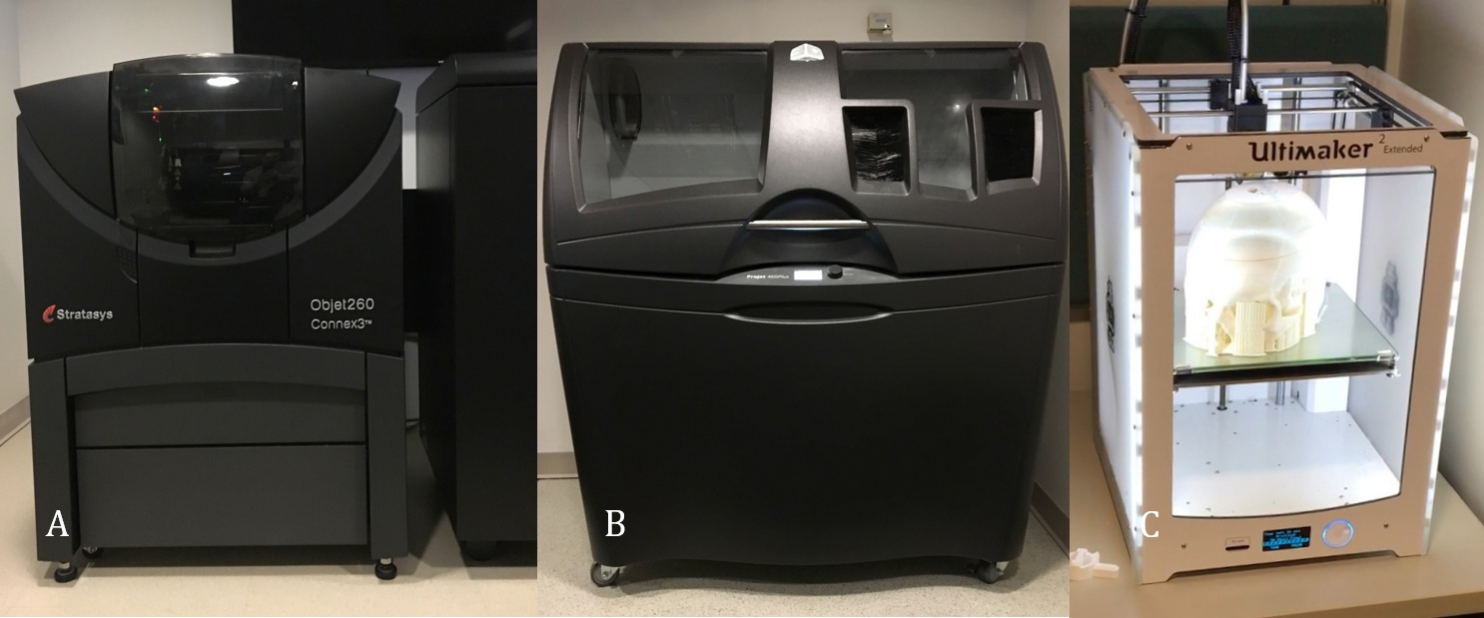
Our institutional printers A) Stratasys Objet 260 Connex 3 (polyjet, photo curable resin); B) 3D systems CJP460 Plus (binder jet, gypsum powder); C) Ultimaker’s 2 and 3 (fused deposition modeling, also known as FDM).

The file is then sent to a 3D printer of choice. There are many different types of 3D printing techniques that will yield very different results. At our institution, our printer array consists of four printers: Stratasys Objet 260 Connex 3 (Polyjet, photo curable resin) (Stratasys Ltd., Eden Prairie, MN) (Figure 3A), 3D Systems CJP460 Plus (Binder jet, gypsum powder) (3D Systems, Rock Hill, SC) (Figure 3B), and Ultimaker’s 2 and 3 (fused deposition modeling, also known as FDM) (Ultimaker, Cambridge, MA) (Figure 3C). The printer is selected based on the clinical need and the application. The printers also complement each other well for a collaborative printing model of different materials and texture.

The Stratasys is capable of producing models using digital materials. This means that several types of resin can be automatically mixed by the printer to create flexible or rigid models. The printer can also produce transparent, opaque, or full-color models. The 3D Systems printer produces rigid, full-color models that feel very much like bone, and the Ultimaker printers produce single material models with generally a rigid structure (there are some semi-flexible experimental materials available).

To produce the most realistic models, we triage the case to a printer that will best replicate the anatomy. If we are printing vasculature or brain tissue, we use the Stratasys printer due to its capability of producing flexible models. If the skull or other bony structures are of interest, the 3D System's printer produces the most realistic bone models. 

Institutional costs

Cost has been a barrier to many institutions due to the necessity of implementing hospital-based printing labs. The hardware, software, and consumables required to produce 3D printed models can be high. The cost of the 3D printed model is variable based on the printer technology used and the size of the model. The same model will cost different amounts to print on each of the three printer types that we own, with the Polyjet being the costliest, and the FDM printers being the cheapest. The Binder jet printer is on the lower side of cost. Typically, a model will cost our institution approximately between $100 and $600, factoring in consumables and labor. That same model outsourced could cost up to a few thousand dollars. The initial outlay of funding for a printing lab is high, but models can be produced at a much lower cost, saving money over time.

Institutional applications of MMTS

**Figure 4:**
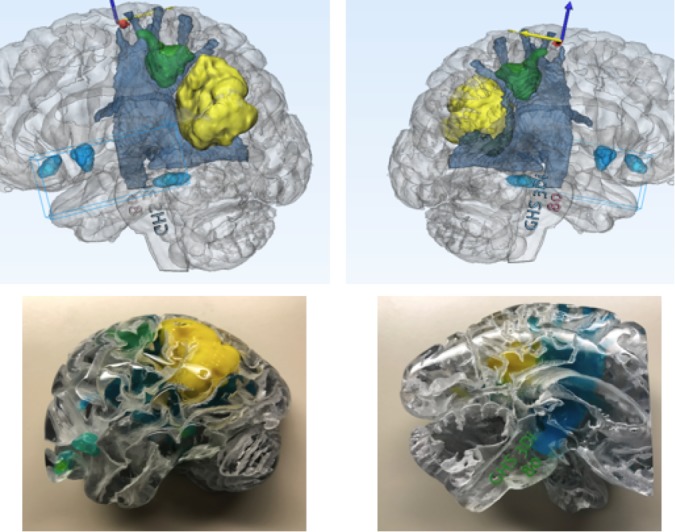
MMTS protocols and semi-automated tractography techniques Production of patient-specific model that displays physical (blue) and functional structures (green) in relation to the lesion of interest (yellow). Blue and yellow arrows are arrows of axes. MMTS: multimodality 3D superposition

**Figure 5:**
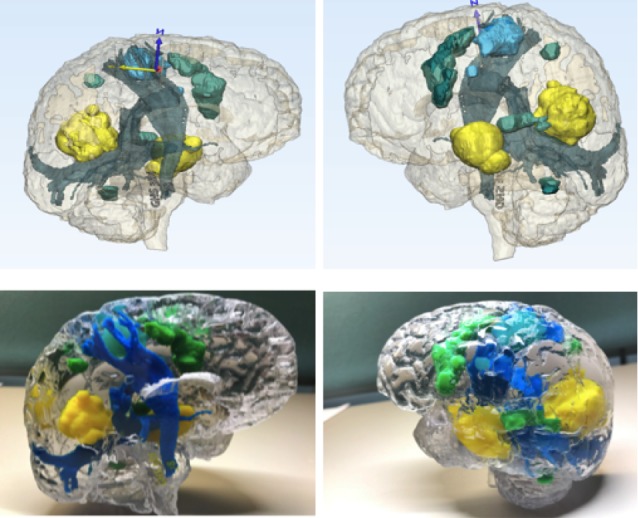
MMTS protocols and tractography Production of a patient-specific model that displays physical (blue) and functional structures (green) in relation to the lesion of interest (yellow). Blue and yellow arrows are arrows of axes. Blue "N" represents "north direction." MMTS: multimodality 3D superposition

With our institutional MMTS protocols and semi-automated tractography techniques, we have printed numerous patient-specific 3D models, two of which are showcased in this report (Figures 4-5). Using this all-inclusive model, which displays pertinent structures in relation to the lesion of interest, gives an overall view and full appreciation of critical functional areas and cerebral connectivity affected by the pathology. We have noted an improved understanding of the patient’s disease, surgical planning, and appreciation for eloquent areas by our residents and surgical staff.

## Discussion

DTI, tractography, and patient outcomes

Since the initial appearance of DTI in the literature in the early 90’s, over 2,000 publications have explored the role of DTI in neuroimaging, most of these within the last decade [4]. DTI has been shown to have significant value in predicting preoperative neurological impairment and postoperative outcomes, as well as assisting with surgical planning across the entire spectrum of neurosurgical diseases. The role of DTI in improving outcomes has been established in both low and high-grade gliomas (HGG), including glioblastomas (GBM), meningiomas, and medulloblastomas. It also serves as an adjunct to the awake craniotomy for tumor resection and radiation planning for stereotactic radiosurgery [5-8].

Gliomas are known to invade not only cortical, but also subcortical structures, and show infiltrative progression along white matter tracts. As such, anatomic fiber tracking and functional mapping of the subcortical white matter tracks is an extremely useful adjunct to achieve maximal safe surgical resection [9]. Reports reveal fewer postoperative motor deficits and higher six-month Karnofsky Performance Scores (KPS) with the use of preoperative DTI and tractography, along with a higher likelihood of achieving gross total resection in patients with HGG [10]. Although without statistical significance, there are also suggestions of improved postoperative morbidity.

Fractional anisotropy has been shown to successfully correlate with neurological impairment and has the most robust evidence for clinical utility, particularly in patients with ischemic or hemorrhagic stroke and cervical spondylotic myelopathy [4]. The number of fibers (NF) index, {healthy corticospinal tract (Hcst) NF - tumoral corticospinal tract (Tcst NF)/(Hcst NF)} (where Hcst NF is the number of fibers calculated for the healthy corticospinal tract (CST) and Tcst NF is the equivalent value for the tumor site), is another valid biomarker that was shown to predict postoperative motor outcomes in patients with low-grade gliomas [11]. These advantages of DTI call for standardized automated tractography to reap the benefits of accurate representations of cerebral connectivity.

Limitations of manual and semi-automated tractography

Although well-established as a promising emerging tool in the neurosurgeon’s armamentarium, the application of semi-automated software and targeting ROIs has its limitations. Using our MMTS technique, we’ve created several 3D printed models fusing our semi-automated deterministic fiber tracking techniques with contrasted T1, fMRI, and CTA/MRA. One concern is that the quality of available DTI data varies between published studies due to the variability among imaging platforms, acquisition methods, post-processing software, and users [11]. An important factor contributing to inter-observer variability is that the selection of ROI cannot be fully automated due to low sensitivity and specificity with institutionally available tools and software. Unblinded manual ROI selection is slow and imprecise and introduces potential bias, as the technician can arbitrarily include or omit pixels of high or low signal. It is also not feasible to blind the technician in many scenarios. A consistent AWBT protocol helps significantly in mitigating these issues.

Another major limitation of manual or semi-automated tractography from DTI is the inaccuracy of detecting fiber tracts in close proximity to or within the tumor on preoperative imaging due to changes in tissues caused by edema, tissue compression, degeneration, or tumor invasion. Intraoperatively, brain relaxation after lesion resection prevents real-time confirmation of fiber tract position; however, this may be offset to some extent by preventive measures (limiting the drainage of cerebrospinal fluid, entry into the lateral ventricle, and brain tissue retraction), intraoperative stimulation mapping of the subcortical pathways, intraoperative imaging, non-rigid registration, and the use of a deformation atlas.

3D printing and patient outcomes

Data on the impact of 3D printing on clinical outcomes in the neurosurgical population, on the other hand, is limited but encouraging. Although long-term outcome data are lacking due to the technology still being in its infancy, patients stand to benefit from its application in neurosurgery. Three-dimensional printing offers significant advantages over conventional methods in preoperative planning and intraoperative navigation. By creating a model of the pathology in question, this technology enhances the surgeon’s understanding of complex anatomy and previously unforeseen challenges. This also creates a patient-specific model, which encourages more fruitful and complete discussions with patients regarding their disease process and upcoming surgeries.

The 3D printed materials can be can be manipulated with surgical maneuvers for cognitive task analysis and also for technical skill rehearsal, making it feasible for surgeons to practice the procedure realistically in an ex vivo environment - performing the surgery before the surgery. As a result, 3D printing has been shown to improve operating time and decrease blood loss, along with a trend towards decreasing surgical site infections (SSIs) [12-13]. A decrease in operative time has been demonstrated in studies predominantly describing 3D models used in orthopedic knee surgeries and maxillofacial surgeries, as well as cranial and spinal surgeries. When used as guides, spinal models for instrumentation and cranial models for deep brain stimulation implants decreased intraoperative fluoroscopy and operative times substantially [12]. SSI is hypothesized to be a result of decreased operating times and blood loss, although conclusive evidence is lacking. Length of hospital stay and risk of other complications has not been demonstrated to be significantly affected by the use of 3D printing and needs further investigation.

Advantages of MMTS

One of the major limitations to tractography is the uncertainty of the degree of anatomic to functional variability and the question of what role patient-specific neuroplasticity plays in redefining conventional dogmas of functional suppositions based on anatomy. Extensive work has been published which investigates anatomic and functional relationships - that is, functional predictability based on structural certainties. More specifically, there has been data to support that combining fMRI and DTI increases functional certainty and underpins the accuracy of the information gained from cerebral connectivity analyses [14-15].

By combining multiple complex imaging sequences, we can display the patient’s pathology in its exact location and critically examine these precise relationships of contrast-enhancing areas, surrounding neurovascular and bony structures, and areas of functionality on top of the tractography printed on physical 3D models (Figures 3-4). This application of MMTS gives the neurosurgeon an indispensable tool that accurately represents both the physical and functional structures related to the patient-specific pathology. With a hand-held 3D model of accurately layered information fused into it, surgical planning has never been so easy.

Three-dimensional printing in neurosurgery has particularly been popular in the neurovascular sector with models printed for aneurysm clipping. Complex as they may be, their natural occurrence in the subarachnoid, extra-axial spaces makes DTI sequences less of a priority. As pathologies are more intimately intertwined within and around eloquent functional zones of the brain, the call for a reliable AWBT in 3D printing becomes more desirable. Large intricate arteriovenous malformations (AVMs) involving, both deep and superficial, eloquent and non-eloquent areas, with previous hemorrhage and surrounding encephalomalacia, are strong applications for AWBT 3D printing not only for surgical planning but also for patient education. To our knowledge, there have not been any reports on superposing various imaging sequences into one comprehensive 3D model for neurosurgical use and valued care improvement. Here, we explain our technique of MMTS, which presents additional pertinent information to the 3D printed patient-specific model, such as functional data from fMRI imaging alongside tractography.

Cost

A potential contributor burdening widespread adoption of 3D printing technology is the cost of acquiring and maintaining the ability to provide this service. Three-dimensional printing devices can be expensive, although their cost has been decreasing recently since most 3D printing systems are now off-patent. Prices range from $2,000 for entry-level FDM, stereolithography (SLA), and PolyJet systems to $150,000 – $900,000 for the most accurate SLS, FDM, SLA, and PolyJet systems. Consumer printers are available at relatively low prices, but the quality of the end product is suboptimal. Software costs range from $500–$10,000 depending on the software package, although free open-source software is available. The cost of materials is also variable, leading to a wide variation in the per unit cost of the production of specific items. As such, the reported per unit costs range between $0.46 per each Army-Navy surgical retractor manufactured with FDM, to $1,200 per stainless steel bone reduction clamp [16].

Contrarily, time saved in the operating rooms (OR) can translate into significant cost savings. In monetary terms, for example, 10 minutes saved in an operating room can potentially have the same value as one hour of work on the object design or its production. This consideration was addressed and accounted for by Lethaus, et al. from Netherlands [17], who estimated the cost of operating time as 16 Euros (€) per minute and the cost of an anatomic 3D model between 200 and 250€. The use of a 3D model was estimated to save an average of 25.2 minutes per procedure, i.e., 403€; thus, the time saved counterbalanced the cost of the 3D model.

Printing costs include money spent on supporting devices, materials, and services in addition to the cost of the initial purchase of the printer. Depending on the needs of an institution, it may be necessary to hire a worker specifically for this purpose. As such, the use of commercial services for 3D printing is not uncommon and can be cost-effective in a low volume setting. With high volume printing, however, it may be prudent to purchase a printing system as per unit costs become more affordable.

Education

**Figure 6:**
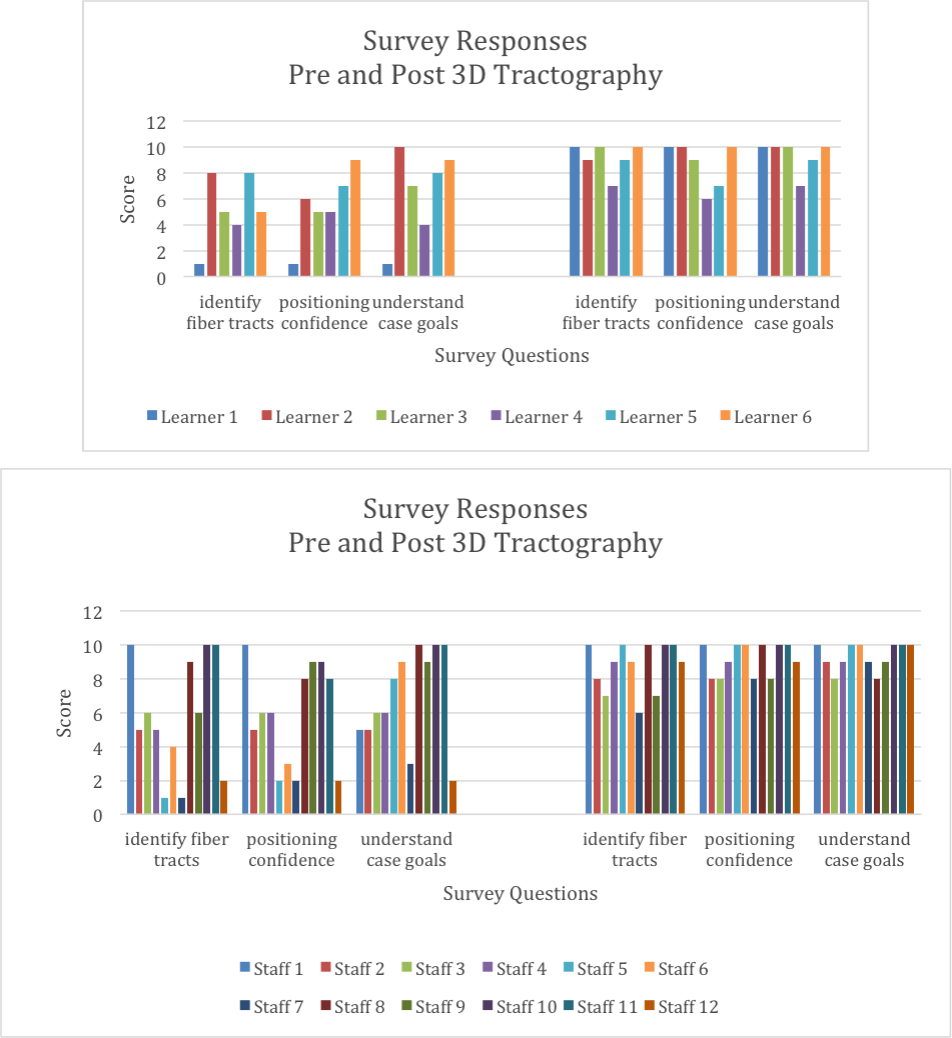
Survey responses before and after 3D tractography model review Six learners and 12 operating room staff were given survey questions before (left) and after (right) reviewing a three-dimensional (3D) tractography model. Scores given ranged from 0 (strongly disagree) to 10 (strongly agree). Identification of fiber tracts, confidence with patient positioning, and understanding of case goals were all significantly improved (p < 0.5) after review of the 3D tractography model.

**Figure 7:**
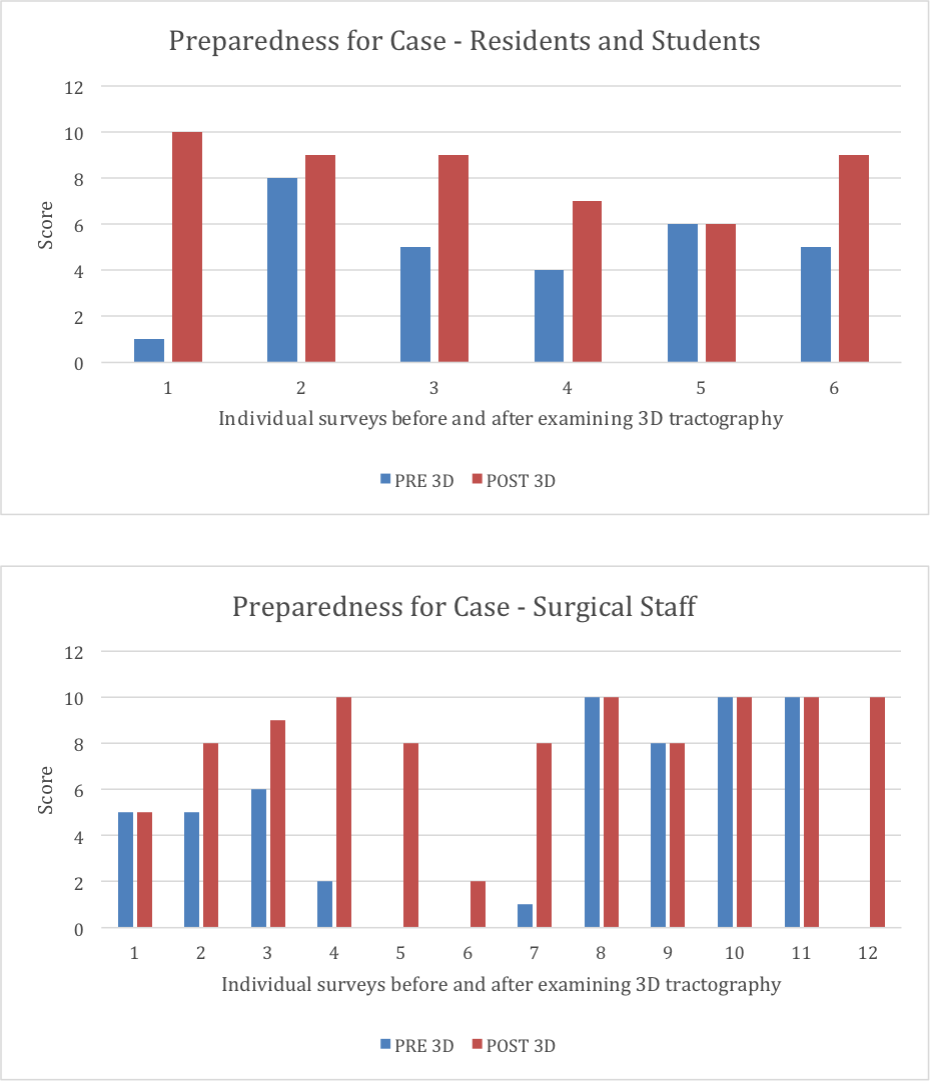
Preparedness for case Six learners and 12 operating room staff were given survey questions before (blue) and after (red) reviewing a three-dimensional (3D) tractography model. Scores given ranged from 0 (strongly disagree) to 10 (strongly agree). Perception of case preparedness was significantly improved (p < 0.5) after review of the 3D tractography model.

The usefulness of 3D printing over other traditional educational models for learning anatomy has been demonstrated [18]. At our institution, we are using MMTS as an educational tool not only for residents and attending physicians but also for supporting staff and, most importantly, for our patients. A research determination worksheet (RDW) was submitted to our IRB. It did not meet the definition of research and was therefore not subject to research regulations. Questions were given to residents/students and surgical staff regarding lesion location, surrounding structures, patient positioning, the importance of structures, fiber tract location, case goals, and case preparedness. Instructions were given to circle a number from a 0-10 scale (with "0" labeled "strongly disagree", "5" labeled "somewhat agree", and "10" labeled "strongly agree") for each question. Surveys completed by resident members and operating room staff within our neurosurgery department revealed an increased understanding of fiber tracts, intraoperative patient positioning, and overall case goals (Figure 6). Both residents and surgical staff members reported they were more prepared for the case compared to how they felt when they did not have a 3D model available (Figure 7). Pre-3D model surveys compared to post-3D model surveys in both residents and surgical staff showed a significant improvement in overall understanding (p = 0.019 and p = 0.014, respectively, p-value of < 0.05 = significance). Moreover, we have demonstrated its utility in preoperative surgical planning, which can have a large impact on workflow and can decrease OR times, and subsequently may have profound influences on reducing the incidence of SSI, decreasing blood loss, and improving outcomes.

Patient communication

With continued efforts on valued care and patient satisfaction, patient communication has never been more important. Our comprehensive patient-specific 3D models produced by MMTS welcome insightful discussion between patients and their surgeons. Without a priori knowledge of CTs or MRIs, the patient can understand their disease process and become engaged in surgical considerations and planning by examining and reviewing their 3D model. These discussions also encourage questions and breaks new barriers in both the inpatient and outpatient settings, creating a new meaning of full, informed consent. With patients being more active participants in their care, this may promote adherence to recommended treatment and decreases the chances of medical errors and the likelihood of malpractice litigation [19].

Future directions

Continued work with 3D printing in neurosurgery and rapid technological advances in AWBT create an exciting future for these applications. Further studies are yet to be reported on comprehensive 3D models with AWBT. This may have the potential of revealing newly defined surgically modifiable risk factors, such as OR time and radiation exposure to improve patient outcomes and inspire patient communication and engagement. The anticipated advantages of our preliminary data and technical report have been strongly supported by previous publications, though validation through our institutional demonstration of decreased OR times, SSI, patient engagement, patient satisfaction, and patient outcomes by using 3D MMTS models generates an intriguing avenue for future publications.

## Conclusions

With numerous advantages to performing tractography from DTI sequences, the demand for accurate and consistent fiber tracking via AWBT is high. With the emergence of 3D printing in neurosurgery, software complexity and equipment availability to institutions should encourage innovative protocols not only to precisely depict lesions in the brain, but also to advance safe practice and research. These developments will also assist with building a well-informed team-based approach to rally patient satisfaction and valued care.
